# ﻿*Lepisorusmedioximus* (Polypodiales, Polypodiaceae), a new species from Shan State of Myanmar

**DOI:** 10.3897/phytokeys.201.84911

**Published:** 2022-06-20

**Authors:** Tao Fujiwara, Phyo Kay Khine, Kiyotaka Hori, Thant Shin, Noriaki Murakami, Harald Schneider

**Affiliations:** 1 Makino Herbarium, Tokyo Metropolitan University, 1-1 Minami-osawa, Hachioji, Tokyo 192-0397, Japan Xishuangbanna Tropical Botanical Garden, Chinese Academy of Sciences Menglun China; 2 Center for Integrative Conservation, Xishuangbanna Tropical Botanical Garden, Chinese Academy of Sciences, ﻿Menglun 666303, China Tokyo Metropolitan University Tokyo Japan; 3 The Kochi Prefectural Makino Botanical Garden 4200-6 Godaisan, Kochi 781-8125, Japan The Kochi Prefectural Makino Botanical Garden Kochi Japan; 4 Forest Research Institute, Yezin, Nay Pyi Taw, Myanmar Forest Research Institute Nay Pyi Taw Myanmar

**Keywords:** Epiphyte, integrative taxonomy, micromorphology, overlooked species diversity, phylogeny

## Abstract

A new species of the species-rich fern genus *Lepisorus* (Polypodiales, Polypodiaceae) has been found to occur in Shan state, Myanmar. *Lepisorusmedioximus* is described based on morphological characters and phylogenetic evidence. Phylogenetic analyses showed that the specimens of *L.medioximus* formed a distinct clade nested in the *Pseudovittaria* clade. The morphological comparison demonstrated that the species is distinct from phylogenetically related species, namely *L.elegans*, *L.contortus*, and *L.tosaensis*, in the morphology of the rhizome scales, size, and shape of the lamina, position of sori, and paraphyses.

## ﻿Introduction

The genus *Lepisorus* (J. Sm.) Ching (Polypodiaceae) occurs throughout Eastern and Southern Asia with range extensions towards the Pacific islands including Hawai’i and towards tropical Afromadagascar (Ching 1933; Zink 1993; Wang et al. 2012). Taking into account the various taxonomic studies and the recent proposal to expand the generic circumscription by including all genera of the tribe Lepisoreae such as *Lemmaphyllum* C. Presl, *Lepidomicrosorium* Ching & K.H. Shing, *Neocheiropteris* Christ, *Neolepisorus* Ching, *Paragramma* (Blume) T. Moore, and *Tricholepidium* Ching (Zhao et al. 2020) *Lepisorus* can be currently recognized as one of the most species-rich genera among genera in Polypodiaceae, comprising ca. 90 species in 18 sections (PPGI 2016; Fujiwara et al. 2020; Zhao et al. 2020). Whereas the core of the genus *Lepisorus* (*Lepisorus* s.s.) is easily recognized by its unique suite of morphological characters including creeping rhizomes covered by clathrate scales, simple leaves, and sori covered with scale-like paraphyses (Ching 1933; Qi et al. 2013; Zhao et al. 2020), some controversy still exists concerning the broader circumscription to avoid the need to recognize the genus *Ellipinema* Li Bing Zhang & Liang Zhang (Zhang et al. 2020). Despite significant progress (Wang et al. 2010a; Wei et al. 2017; Zhang et al. 2020; Zhao et al. 2020), taxonomic uncertainty is arguably not restricted to the generic classification but affects the estimation of the total species diversity that is expected to be still underestimated due to the difficulty in the taxonomic classification of this genus. Several characters utilized as key information on species delimitation show high variation within some species (Wang et al. 2010b). In particular, the identification of species relies on a few diagnostic characters such as the shape of the lamina, position of sori, and structure of the rhizome scales and paraphyses. Unfortunately, these characters are hardly diagnosable in the field. As a consequence, some species have been frequently misidentified or overlooked, as exemplified by the recent reclassification of Japanese *L.thunbergianus* (Kaulf.) Ching and relatives (Fujiwara et al. 2018), and the rediscovery of *Lepisoruscespitosus* Y.X. Lin previously known only as type specimens (Fujiwara et al. 2020).

Yunnan has been increasingly recognized as the diversity hotspot of *Lepisorus* s.s (Wang et al. 2012; Fujiwara et al. 2020). While sufficient explorations of the *Lepisorus* diversity have been made available for China and India, this cannot be claimed for the regions south and southwest of Yunnan, namely Laos and Myanmar. These two countries as well as Thailand show extremely low species diversity of the genus despite being expected to harbor a notable diversity of this genus (Suppl. material 1: Table S1). This can be attributed to not only the difficulty in the taxonomic classification of this genus but also the underestimation of whole fern species diversity due to fewer flora surveys previously conducted. Thus, overlooked species are expected to occur in these regions.

The Shan state of Myanmar is the focus of this study. The Shan state covers 155,800 km^2^ which is almost a quarter of the whole area of Myanmar and is mainly comprised of a hilly plateau bordering Yunnan, China in the north, Laos in the east, and Thailand in the south. Shan state has been in historical times much less surveyed than Yunnan Province of China although the latter is known for its rich diversity of ferns including *Lepisorus*. Thus, we expect to retrieve not only new records but also some new fern species that are putative endemics to Shan State. To make this expectation tested, floristic inventories were carried out across the Shan state in September 2019. Two unusual specimens of *Lepisorus* were collected in Pin Laung Township, Ka Thaung (upper) located in the southern part of the state, which were recognized as a putative new species. This proposal was studied by consulting checklists of Myanmar and adjacent areas (Dickason 1946; Lindsay and Middleton 2012; Qi et al. 2013; Khine et al. 2017; Khine and Schneider 2020; Hori 2021; Vongthavone et al. 2021), and careful comparison of morphological characters with previously described species by consulting specimens and species protologues (e.g., Ching 1933; Bir and Trikha 1969; Yu and Lin 1996, 1997; Lin 2000; Qi et al. 2013). Besides morphological diagnostics, we employed DNA sequences to identify genotypic evidence supporting these two specimens as distinct species from any other species that are previously described.

## ﻿Materials and methods

### ﻿Morphology

The morphology of the two specimens of *Lepisorus* sp. (Hori et al. 108225 and 108229) was compared to descriptions and specimens of species sharing similarities in the main diagnostic features, namely rhizome scales, the size and shape of the lamina, the position of sori, and paraphyses. The morphological observation was conducted using a stereomicroscope. Voucher specimens were deposited in MBK, HITBC, and RAF.

### ﻿DNA extraction and chloroplast DNA region sequencing

Total DNA for each of the two specimens was extracted from silica dried leaves using the CTAB method according to Doyle and Doyle (1987). Four plastid regions, *rbcL* gene, *rbcL-atpB* intergenic spacer, *rps4-trnS* intergenic spacer, and *trnL-trnF* region including the *trnL* intron and the *trnL-trnF* intergenic spacer were amplified according to the protocol provided (Wang et al. 2010b) using ExTaq (TaKaRa Bio Inc., Shiga, Japan). The PCR products were purified using Illustra ExoStar 1-Step (GE Healthcare, Wisconsin, USA) and used as templates for Sanger sequencing. Reaction mixtures for sequencing were prepared using the SuperDye v3.1 Cycle Sequencing Kit (ADS). The reaction mixtures were analyzed using an ABI 3130 Genetic Analyzer (Applied Biosystems).

### ﻿Phylogenetic analyses

To unveil the phylogenetic position of the new *Lepisorus*, a genus-level phylogeny was reconstructed incorporating a total of 88 species of *Lepisorus* including species representing clades previously treated as distinct genera, namely *Lemmaphyllum*, *Lepidomicrosorium*, *Neolepisorus*, *Neocheiropteris*, *Paragramma*, and *Tricholepidium*, retrieved from the sequence matrices assembled in previous studies (Wang et al. 2010b; Fujiwara et al. 2018, 2020; Zhao et al. 2020) (Suppl. material 2: Table S2). *Leptochilusellipticus* (Thunb.) Noot., *Microsorumpunctatum* (L.) Copel., and *Bosmaniamembranacea* (D.Don) Testo were included as outgroup taxa. Sequences of each plastid region were separately aligned using MAFFT (Katoh and Standley 2013) followed by manually editing in Aliview (Larsson 2014), and subsequently merged into a concatenated matrix using SequenceMatrix (Vaidya et al. 2011). GTR +I +G was adopted as the best substitution model as selected based on AIC using jModelTest 2.1.10 (Darriba et al. 2012). Phylogenetic hypothesis reconstruction was performed with three different methods: maximum likelihood (ML), Bayesian inference (BI), and maximum parsimony (MP). ML analyses were performed using IQ-TREE v.1.6 (Nguyen et al. 2015) with default settings. 1000 ultrafast bootstrap replicates were conducted to infer node robustness (Hoang et al. 2018). For the BI method, MrBayes 3.2.6 (Ronquist et al. 2012) was employed by applying two runs of four MCMC chains for 100,000,000 generations with samples taken every 1000 generations. Tracer 1.6 (Rambaut and Drummond 2013) was used to evaluate the samples trees with a focus on convergence. The first 25% were discarded as burn-in. The MP analysis was performed using a heuristic approach with TBR branch-swapping, as implemented in MEGAX (Kumar et al. 2018). Ten initial trees were generated by the addition of randomly selected sequences. The robustness of each branch was assessed by bootstrap analysis calculating 1000 replicates.

## ﻿Results and discussion

**Figure 1.**
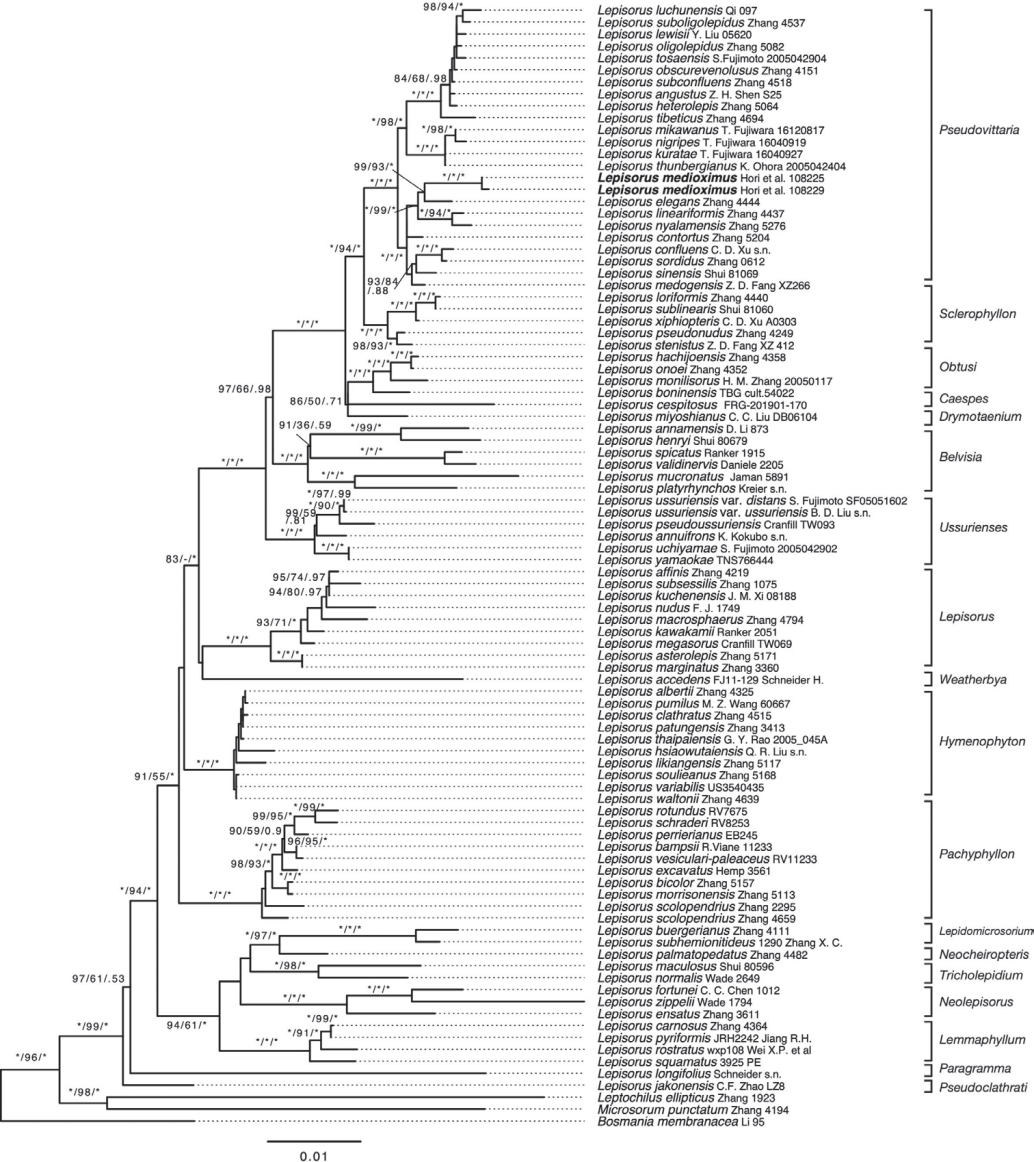
Maximum likelihood tree of *Lepisorus* based on the combined dataset of *rbcL*, *rbcL-atpB*, *rps4*-*trnS* and *trnL*-*F.* The number on each branch indicates support values as follows: ML bootstrap support/MP bootstrap support/BI posterior probability. The classifications of genus and section for *Lepisorus* follows Zhao et al. (2020) and Fujiwara et al. (2020).

The combined dataset of *rbcL*, *rbcL-atpB*, *rps4-trnS*, and *trnL-F* contained 4,617 bp of which 744 sides were parsimoniously informative. The optimal log-likelihood for the reconstructed phylogeny inferred by the ML method was ln = -21430.138. The topologies were congruent among the phylogenetic hypothesis obtained using the three distinct phylogenetic inference approaches. The result showed that two specimens of *Lepisorus* from the Shan state of Myanmar formed a clade with a bootstrap value of 100% (ML ultrafast bootstrap value = 100%, MP bootstrap value = 100%) and BI posterior probability of p = 1.0. This clade was nested in the subclade of the sect. Pseudovittaria clade (Fig. 1) (Zhao et al. 2020) that included *L.contortus* (H. Christ) Ching, *L.elegans* Ching & W.M.Chu, *L.lineariformis* Ching & S. K. Wu, and *L.nyalamensis* Ching & S. K. Wu. While the latter two species were highly distinct from the new species in their linear to linear-lanceolate lamina, the accumulated substitution event causing a rather long branch separated the two specimens from *L.elegans*—the morphologically most similar species of this clade (Fig. 1).

**Figure 2.**
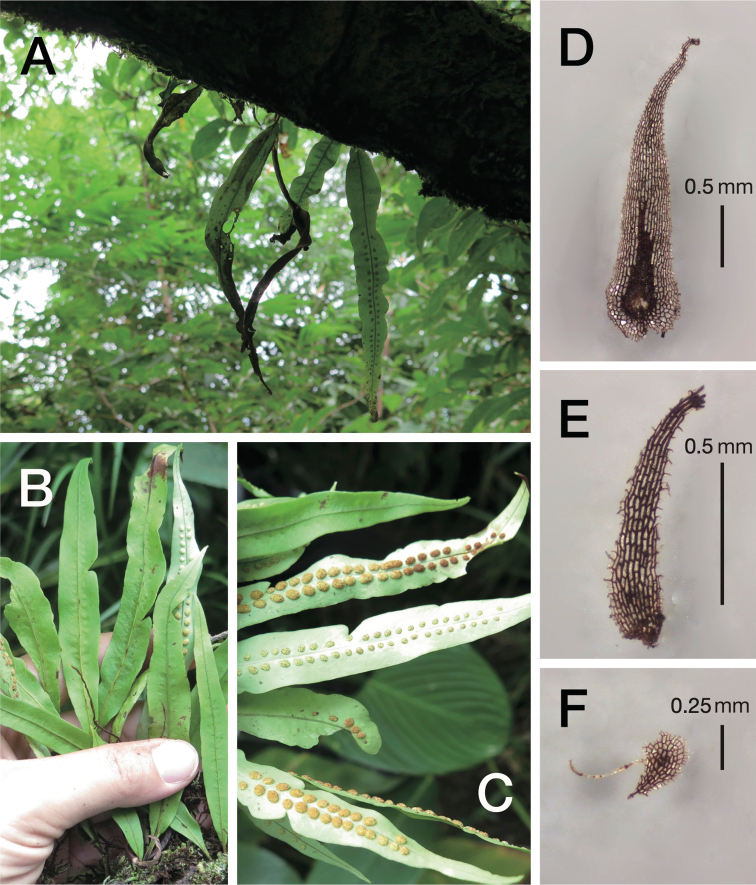
Living plant of *Lepisorusmedioximus* T.Fujiw., K.Hori & Khine **A** habit **B** adaxial side of lamina **C** abaxial side of lamina with sori **D** rhizome scale **E** leaf scale **F** paraphyses.

The morphological comparison revealed that the new species was similar to *L.elegans*, *L.contortus*, and *L.tosaensis* (Makino) H. Itô, species from the sect. Pseudovittaria, consistent with the result of phylogenetic analyses. However, the species was discernible from the similar species in lanceolate, pale brown rhizome scales, very short stipe, lanceolate lamina widest at the proximal 1/3, sori located closely to the costa and restricted to the 3/4 distal part of the lamina, and ovate-lanceolate paraphyses (Table 1 and Fig. 2).

**Table 1.** Comparison of morphological characters between *Lepisorusmedioximus* and three related species.

	*Lepisorusmedioximus*	*Lepisourselegans*	*Lepisourscontortus*	*Lepisorustosaensis*
**Rhizome scale**	Lanceolate, pale brown, iridescent, clathrate with short and narrow, dark brown opaque band, margin entire to subentire, lumina large	Lanceolate, yellow-brown, iridescent, almost clathrate, sometimes with narrow, brown opaque band, margin entire to subentire, lumina large	Broadly lanceolate, pale-brown, clathrate with narrow, brown opaque band, margin denticulate, lumina small	Lanceolate or broadly lanceolate, iridescent, opaque dark brown with clathrate margin, lumina small
**Fronds**	Remote, 0.5–1.5 cm apart	Remote, 0.5–2 cm apart	Remote, 0.5–2 cm apart	Fronds clustered
**Stipe**	Stipe short, straw-colored to deep brown, 0.4–0.8 cm long	Stipe straw-colored to deep brown, 1–5 cm long	Stipe normally straw-colored, less often brown, 2–5 cm long	Stipe straw-colored, 1–3 cm long
**Laminae**	Lanceolate, widest at the proximal 1/3 of lamina, base cuneate, slightly decurrent, apex long caudate	Lanceolate, widest at middle, base cuneate, slightly decurrent, apex long caudate	Linear-lanceolate to lanceolate, widest at middle, base cuneate, decurrent, apex shortly acuminate	Lanceolate to broadly lanceolate, widest at middle, base cuneate, decurrent, apex acuminate
**Leaf scale**	Lanceolate, brown, clathrate	Lanceolate, brown, clathrate	Ovate, pale brown, clathrate	Ovate, brown, clathrate
**Sori**	On distal 3/4 of lamina, close to costa, orbicular to ovate	Restricted to distal 1/3–1/2 of lamina, midway between costa and margins, orbicular	Restricted to distal half, slightly closer to costa, orbicular, or slightly ovate	Restricted to distal half of lamina, close to costa, orbicular
**Paraphyses**	Ovate-lanceolate, ovate to orbicular, brown, clathrate, lumina large, margin entire	Orbicular, brown, lumina small, usually opaque, sometimes clathrate, margins with awn-spines	Orbicular, brown, clathrate with center dark brown, thick and opaque, margin denticulate	Orbicular, brownish, clathrate, central lumina small, margin denticulate

### ﻿ Lepisorus medioximus

Taxon classificationPlantaePolypodialesPolypodiaceae

T.Fujiw., K.Hori & Khine sp. nov.

5281EF09-1DA8-5F49-939A-733787100598

urn:lsid:ipni.org:names:77300053-1

Figs 2 , 3

#### Diagnosis.

The new species differs from similar species, *Lepisoruselegans* and *L.contortus*, by the combination of the following morphological characteristics: the lanceolate laminae with the widest at proximal 1/3 of the lamina, sori closer to costa, sori on distal 3/4 of the lamina, and ovate-lanceolate, ovate to orbicular clathrate paraphyses with entire margins. The species is discernible from *L.tosaensis* by pale-brown lanceolate rhizome scale with a narrow opaque band, remote fronds, and lanceolate leaf scales.

#### Type.

Myanmar. Shan state: Pin Laung Township, Ka Thaung upper, 19°57'58.5"N, 96°31'09.1"E, alt. ca. 904 m, 26 Sep. 2019, K. Hori, P.K. Khine [“Kine”], T. Fujiwara, M. Nagashima, P.P. Shwe & A.K. Moe 108225 (holotype: MBK0328223!, isotype: HITBC! and RAF!).

#### Description.

Plant epiphytic. Rhizomes long creeping, 0.10–0.15 cm in diam., densely scaly, sometimes naked when old; Rhizome scales lanceolate, pale-brown, iridescent, clathrate with short and narrow, dark brown, opaque center band, 2.1–2.8 mm long × 0.4–0.6 mm wide, margin entire to subentire, apex acuminate, lumina large. Fronds remote, up to 1.5 cm apart; stipe short, 0.4–0.8 cm long, 0.6–1.0 mm diam., straw to dark brown colored; Lamina lanceolate, abaxially grayish-green, adaxially light green when fresh, 8–16 cm long × 0.9–1.5 cm wide, widest at proximal 1/3 of lamina, thinly leathery, adaxially glabrous, abaxially sparsely scaly, lamina base attenuate, decurrent, apex long caudate; costa raised on both sides, veinlets obscure; Leaf scales lanceolate, brown, clathrate, 0.8–1.4 mm long × 0.1–0.3 mm wide, margin denticulate, apex acuminate; Sori on distal 3/4 of lamina, very close to costa, orbicular or elliptic, 0.17–0.35 mm long × 0.12–0.23 mm wide, occasionally sunken on abaxial side of lamina; Paraphyses ovate-lanceolate, ovate to orbicular, brown, clathrate, lumina large, margin entire, 0.19–0.28 mm in diam.

#### Etymology.

The epithet ‘medioximus’ refers to the sori attached to the middle location on lamina.

#### Distribution.

This species is only known from the type locality in Myanmar, Shan state.

#### Habitat.

Epiphyte on tree trunks and branches in evergreen to sub-evergreen forest.

#### Additional specimens examined (paratypes).

Myanmar. Shan state: Pin Laung Township, Ka Thaung upper, 19°57'58.5"N, 96°31'09.1"E, alt. ca. 904 m, 26 Sep. 2019, K. Hori, P.K. Khine [“Kine”], T. Fujiwara, M. Nagashima, P.P. Shwe & A.K. Moe 108229 (MBK 0328227!, HITBC! and RAF!).

#### Note.

Until now, we have not discovered additional specimens from other localities despite our exhaustive search focusing on herbarium specimens collected in all parts of Myanmar and the Yunnan province of China. We specifically checked not only the Myanmar *Lepisorus* specimens deposited to the Makino Botanical Garden (MBK), the Institute of Botany, Chinese Academy of Sciences at Beijing (PE) and the Royal Botanic Gardens (K) but also the *Lepisorus* specimens of Dickason collection deposited in the United States National Herbarium (US), the Natural History Museum (BM), and Naturalis Biodiversity Center (L). Given the observation of more than 50 individuals of the species at the type locality, we expected this species to be abundant in this poorly collected area. Further inventories in Shan state and the adjacent areas should be necessary to find new localities of the species and evaluate the conservation status of the species. Reflecting our limited knowledge, the IUCN red list status of this species is given as “Data Deficient” instead of “Critical Endangered”. The latter status would assume a restriction of this species range to the two localities recorded.

**Figure 3.**
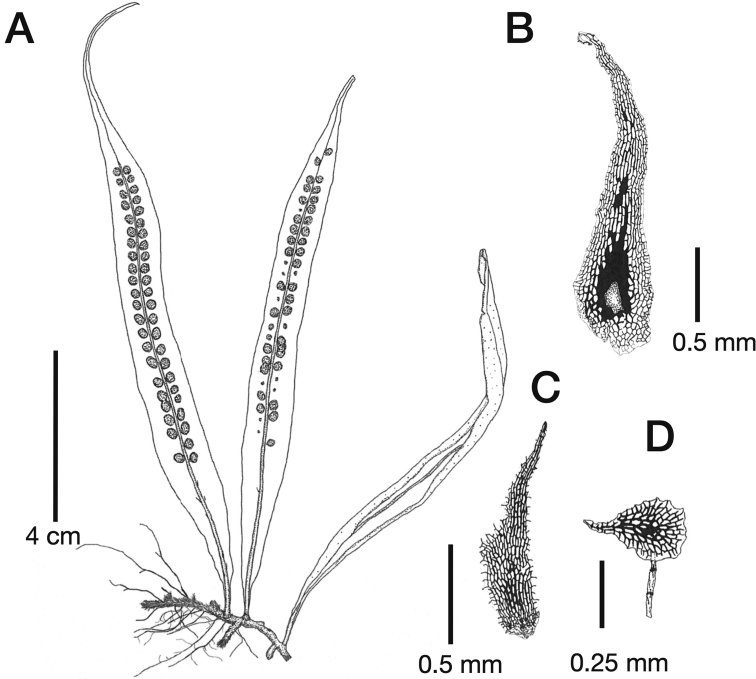
*Lepisorusmedioximus* T.Fujiw., K.Hori & Khine (holotype, Hori et al. 108225 = MBK0328223) **A** habit **B** rhizome scale **C** leaf scale and **D** paraphyses.

## Supplementary Material

XML Treatment for Lepisorus medioximus

## Supplementary materials

Supplementary material 1 Table S1 Data type Excel datasheet Explanation note A checklist for *Lepisorus* s.s. (sensu stricto) species occurrence in Yunnan and the adjacent regions of Indochina. https://binary.pensoft.net/file/703783This dataset is made available under the Open Database License (http://opendatacommons.org/licenses/odbl/1.0/). The Open Database License (ODbL) is a license agreement intended to allow users to freely share, modify, and use this Dataset while maintaining this same freedom for others, provided that the original source and author(s) are credited.Tao Fujiwara, Phyo Kay Khine, Kiyotaka Hori, Thant Shin, Noriaki Murakami, Harald Schneider

Supplementary material 2 Table S2 Data type Excel datasheet Explanation note Summary table for the information of species used in the phylogenetic analyses in this study. https://binary.pensoft.net/file/703784This dataset is made available under the Open Database License (http://opendatacommons.org/licenses/odbl/1.0/). The Open Database License (ODbL) is a license agreement intended to allow users to freely share, modify, and use this Dataset while maintaining this same freedom for others, provided that the original source and author(s) are credited.Tao Fujiwara, Phyo Kay Khine, Kiyotaka Hori, Thant Shin, Noriaki Murakami, Harald Schneider
